# Influence of Education Status, Age, and Gender on HIV Prevention Awareness and Preferences Among Youth in a rural Ugandan district: A Cross-Sectional Study

**DOI:** 10.21203/rs.3.rs-5543904/v1

**Published:** 2025-04-09

**Authors:** Paul Waswa Ssali, Timothy Mwanje Kintu, Tonny Kyagambiddwa, Immaculate Karungi, Agnes Kisakye Namuyaba, Ruth Namaseruka, Mark Agaba, Celestino Obua, Edith K Wakida, Jerome Kahuma Kabakyenga

**Affiliations:** Mbarara University of Science and Technology; Mbarara University of Science and Technology; Mbarara University of Science and Technology; Mbarara University of Science and Technology; Mbarara University of Science and Technology; Mbarara University of Science and Technology; Mbarara University of Science and Technology; Mbarara University of Science and Technology; Mbarara University of Science and Technology; Mbarara University of Science and Technology

**Keywords:** HIV prevention, youth, education status, age, gender, pre-exposure prophylaxis

## Abstract

**Background:**

Young people aged 15–24 years continue to be disproportionately affected by HIV globally. In sub-Saharan Africa, utilization of HIV prevention services remains suboptimal. Education plays a pivotal role in enhancing HIV prevention knowledge among youth, but its effectiveness is influenced by factors such as age, gender, and socioeconomic status. The objective of this study was to investigate how education status, age, and gender influence awareness use, and preferences of HIV prevention methods among youth in rural southwestern Uganda.

**Methods:**

A cross-sectional quantitative study was conducted in March 2022 among 216 adolescents and young adults aged 15–24 years in Rubirizi District, southwestern Uganda. Participants were recruited using stratified random sampling from three publicly funded schools and snowball sampling from local trading centers to include both school-going and non-school-going youth. Data were collected using a structured questionnaire translated into the local language. Descriptive statistics and inferential analyses were performed using R software, with statistical significance set at p < 0.05.

**Results:**

A majority (72%) perceived themselves at low risk of HIV infection, with school-going youth demonstrated higher awareness of abstinence (75% vs. 32%, *p* < 0.001) and prevention of mother-to-child transmission (PMTCT) (46% vs. 25%, *p* = 0.002) compared to non-school-going youth. Younger individuals were more likely to practice abstinence (mean age 18.9 years vs. 19.7 years, *p* = 0.017). Males also had higher awareness of being faithful (59% vs. 44%, p = 0.04). Private doctors (46%) and hospitals (47%) were the most favored access points for PrEP. Younger participants expressed greater concern about daily pill-taking inconvenience (mean age 20.4 years vs. 19.2 years, p = 0.04). Radio (73%) and television (41%) were the most preferred methods for HIV information dissemination. Females preferred magazines more than males (21% vs. 10.3%, p = 0.046).

**Conclusion:**

Education status, age, and gender influence HIV prevention awareness through differences in access to information, risk perception, and preference of prevention methods among youth in rural Uganda. Therefore, aligning HIV prevention strategies with the specific needs and preferences of different youth subgroups can enhance awareness, risk perception, and utilization of HIV prevention methods.

## Introduction

Globally, in 2022, over 410,000 young people aged between 15–24 years were newly infected with HIV[1] accounting for about 27% of all new infections[2]. And in 2023, there were an estimated 3.1 million individuals of the same age group living with HIV/AIDS[3], highlighting the vulnerability of this demographic. Sub-Saharan Africa still bears a disproportionate share of this burden, with HIV/AIDS remaining a major driver of mortality among young people in the region[4]. Recognizing this significant impact, the Joint United Nations Program on HIV/AIDS (UNAIDS) has emphasized the need for comprehensive national prevention strategies that holistically address the diverse needs of young people, particularly in high-incidence populations[5]. This directly aligns with its Sustainable Development Goal 3 which emphasizes promoting well-being among all age groups including ending the AIDs epidemic by 2030[6]. Despite global efforts to curb the epidemic, utilization of HIV prevention services among youth remains suboptimal, especially in Uganda where 40–50% of new HIV infection occur in individuals aged 15 to 24 years [7, 8]. A considerable number of young people are not adequately linked to essential services, leading to continued transmission and new infections.

Education is often a pivotal factor in enhancing HIV prevention knowledge and promoting healthy behaviors among youth[9–11]. Schools play a key role in sharing information about HIV transmission and prevention strategies, covering both conventional methods such as abstinence, being faithful, and condom use, and biomedical interventions like pre-exposure prophylaxis (PrEP), post-exposure prophylaxis (PEP)[12]. Educated individuals are typically more engaged in HIV education programs and better equipped to use prevention services[13]. However, youth not in school may lack access to this information[14], increasing their risk of HIV infection. Nonetheless, the evidence about the connection between level of education and HIV prevention is not always straightforward[15–17], as effectiveness not only depends on the availability of information but also on how it is delivered, received, and applied within different contexts.

Other factors such as age, gender, socioeconomic status, and access to tailored health communication channels may also significantly influence HIV awareness and prevention. Younger individuals may lack exposure to accurate information, while older youth might have more access to nuanced prevention strategies[18]. Gender norms can restrict access to health information, especially for young women while lower socioeconomic status often limits access to prevention methods like condoms, or PrEP [19, 20]. Geographic location also matters, with rural youth having fewer healthcare resources and prevention programs compared to their urban counterparts. Additionally, access to health communication channels, such as public health campaigns, social media[21], and community outreaches, is crucial, as youth disconnected from these platforms may miss out on essential HIV prevention information[22]. These overlapping factors highlight the need to address more than just formal education in efforts to enhance HIV prevention among young people.

Uganda achieved the 90-90-90 goals by 2020, and ongoing efforts are now focused on the 95-95-95 targets, as outlined in the National HIV and AIDS Strategic Plan (NSP) for 2020/2021–2024/2025. The NSP prioritizes implementing combination HIV prevention interventions tailored to specific population groups, while promoting social enablers for equitable access to prevention[23]. However, challenges like low condom use, declining age at sexual debut, and high-risk sexual behaviors among others threaten these achievements [24]. Furthermore, stagnation in HIV knowledge among youth underscores the necessity of youth-focused interventions that align with global frameworks. Rubirizi District in southwestern Uganda, with an HIV prevalence of 7.1%[25], presents a unique context for exploring these dynamics.

As a rural area where HIV incidence is particularly high[26], the district’s demographic and socioeconomic characteristics offer an opportunity to examine how education status, age, and gender influence awareness and use of HIV prevention methods, beliefs about HIV infection risk, preferred access points for PrEP, and effective communication methods for disseminating HIV-related information. Understanding these factors is crucial for developing targeted interventions that address gaps in awareness and utilization of HIV prevention services among different subgroups of youth. Therefore, the purpose of this study was to investigate how education status, age, and gender influence awareness and use of traditional and biomedical HIV prevention methods, beliefs about HIV infection risk, preferred access points for PrEP, and preferred communication channels among youth aged 15 to 24 years in Rubirizi District, Uganda.

## Methods

### Study design and setting

This cross-sectional quantitative study was conducted in March 2022 in Rubirizi district, a rural district in South-western Uganda, previously reported to have a high prevalence of HIV[27]. A standardized questionnaire adapted from Shamu et al[21] was used to investigate how education status, age, and gender influence awareness and use of HIV prevention methods, beliefs about HIV infection risk, preferred access points for pre-exposure prophylaxis (PrEP), and effective communication methods for disseminating HIV-related information among individuals.

### Study population

The study population included adolescents and young adults aged 15 to 24 years in Rubirizi district. Non-school-going youth were defined as those who had less than seven years of formal education, regardless of how long they had spent out of school. The method for sample size estimation for comparative studies by Sharma et al. [28] was used to estimate the sample size; where p1 was 0.3 (1 in 3 youth is known to have knowledge on HIV/AIDS prevention strategies), p2 was 0.5 (since the proportions of non-school-going youth with knowledge HIV prevention services in the population is not known) and c = 7.85, where α = 1.96 (for 95% confidence interval and β = 0.84 (for 80% power). The calculated sample size was 90. In order to cater for non-respondents, 20% of the calculated sample size was added. The final calculated sample size for each stratum was 108 respondents, yielding a total sample of 216. Participants were recruited from: 1) three publicly funded schools where participants were randomly chosen from each class in the school while ensuring to have an equal number of male and female participants in the study; 2) the trading centers in the local communities surrounding the schools with the guidance of a village health worker and snowball sampling. Whereas snowball sampling is a non-probabilistic method, it was used for this population because it was hard-to-reach.

### Data collection procedures

Data was collected using a structured questionnaire with closed end questions that was adopted from Shamu et al. Modifications were made to address the study objectives and fit within the study setting (refer to Additional file 1). The questionnaire assessed HIV prevention awareness by evaluating participants’ knowledge of various HIV prevention methods, including condom use, abstinence, being faithful to one partner, reducing the number of sexual partners, avoiding contact with blood, prevention of mother-to-child transmission (PMTCT), male circumcision, and the use of biomedical methods such as PrEP, PEP, and microbicides.

HIV prevention preferences were measured by exploring perceived risk of HIV infection and reasons for low perceived risk, such as abstinence, condom use, being faithful, and knowing one’s HIV status. Additionally, participants were asked about their preferred access points for PrEP, including private doctors, hospitals, clinics, pharmacies, schools, and community health workers, as well as barriers to PrEP uptake, such as concerns about side effects, lack of knowledge, stigma, and perceived behavioral changes. The study also examined preferred sources of HIV-related information, such as radio, newspapers, SMS, social media, and television, to understand how different demographic groups receive and engage with HIV prevention messaging.

Pre-testing of the tool was done among 12 secondary school students at a school in a neighboring district. While this validated the tool for use among school-going youth, additional contextual adjustments were made to ensure relevance for non-school-going participants. The questionnaire was then translated to Runyankole, the mostly commonly used local language in the area and a printed version used for data collection. All research assistants had a health care background and were trained on how to administer the tool before commencing collection of data.

### Data analysis and management

At the end of each data collection day before leaving the field, the data collection tools were reviewed for completeness by the research assistants. Data was then entered in Kobo Toolbox (Harvard Humanitarian Initiative, Cambridge, Massachusetts, United States of America) [15], an online data collection tool from which an excel sheet was extracted and partially cleaned.

Data were then analyzed using descriptive and inferential statistical methods to explore the impact of education status, age, and gender on various aspects of HIV prevention and communication. Descriptive statistics, including frequencies, percentages, means, and standard deviations, were calculated to summarize the awareness of HIV prevention methods, beliefs about HIV infection risk, preferred access points for PrEP, and preferred communication channels. Chi-square tests were conducted to compare categorical variables such as the knowledge of specific HIV prevention methods, perceptions of HIV risk, preferences for PrEP access points, and communication methods across groups defined by education status (school-going vs. non-school-going), gender (male vs. female), and different age categories. Independent samples t-tests were used to compare mean ages between groups exhibiting different behaviors and beliefs, such as those practicing abstinence versus those who do not, and individuals with low versus high perceived risk of HIV infection. All statistical tests were conducted at a 95% confidence level, with p-values below 0.05 considered statistically significant. Data analysis was carried out using R.

## Results

### Characteristics of study participants

The study enrolled 216 participants,, with a mean age of 19.28 ± 2.32 years, consisting of 50.46% females (n = 109) and 49.54% males (n = 107) and equal proportions of school going and non-school going youth.. All the school going youth reported that they were not in any relationship. The commonest forms of media used as a source of information were radio and television. Of the 216 participants, 173 (80%) reported having previously done an HIV test with 97 (56.1%) being non-school going youth. Only 95 participants (43.9%) reported having heard of Pre-exposure Prophylaxis (PrEP). Participants reported that the three most important things they would want to know about PrEP before using it were: side effects (72.7%), place where they can get the pill (40%) and duration of taking the pill (29%) (Table 1).

### Awareness of different HIV prevention methods

The study revealed that the most known HIV prevention methods among participants were condoms (70%), abstinence (53%), and being faithful to one partner (51.4%). In contrast, less commonly known methods included Post-Exposure Prophylaxis (PEP) at 10%, Pre-Exposure Prophylaxis (PrEP) at 13%, and a vaginal ring at 10%. Education status significantly influenced the awareness of various HIV prevention strategies. School-going individuals exhibited a higher knowledge of avoiding contact with blood (57%) compared to non-school-going individuals (25%, p < 0.001). Additionally, awareness of abstinence was markedly greater among school-going youth (75%) than their non-school-going counterparts (32%, p < 0.001). Similarly, knowledge of Prevention of Mother-To-Child Transmission (PMTCT) was higher in the school-going group (46% vs. 25%, p = 0.002), as was awareness of male circumcision as a prevention method (34% vs. 18%, p = 0.008). Age also played a role in HIV prevention behaviors, with younger individuals more likely to practice abstinence, evidenced by a mean age of 18.9 years compared to 19.7 years for those less likely to abstain (p = 0.017). Gender differences were observed in the understanding of specific prevention methods; males were more likely to be aware of the importance of being faithful (59% vs. 44%, p = 0.04) and PMTCT (44% vs. 30%, p = 0.02) compared to females (Table 2).

### Beliefs about HIV Infection risk

Majority of respondents (72%) were confident that they would not contract HIV, primarily attributing their low perceived risk to abstinence (52.3%) and condom use (37%). Education status significantly influenced these perceptions; non-school-going individuals were more likely to perceive their risk as low due to being faithful to a partner (48% versus 17%, p < 0.01) and the use of condoms (50% versus 30%, p = 0.02). In contrast, school-going individuals frequently cited abstinence as a factor in their low perceived risk of HIV infection (66% versus 26%, p < 0.01). Age also played a critical role in shaping risk perceptions. Younger individuals were more inclined to practice abstinence (mean age 18.3 years versus 19.4 years, p = 0.002), remain faithful (mean age 19.5 years, p = 0.012), know their HIV status (mean age 19.5 years versus 18.6 years, p = 0.022), and avoid sex with sex workers (mean age 17.9 years versus 19.0 years, p = 0.026), all contributing to a lower perceived risk of HIV. Conversely, older individuals were more likely to report having multiple sexual partners (mean age 21.4 years versus 19.9 years, p = 0.02) and a lack of trust in their partners (mean age 22.0 years versus 20.0 years, p = 0.007), which were associated with a higher perceived risk of HIV infection. Additionally, gender differences were evident; males were more likely to avoid sex with sex workers (20% versus 6%, p = 0.02) and use condoms (46.1% versus 28%, p = 0.03) compared to females (Table 3).

### Preferred access points and barriers to use of PrEP

The majority of participants preferred accessing PrEP through private doctors (46%) and hospitals (47%), whereas family members (1%) and community health workers (4%) were the least favored options. The primary perceived barriers to PrEP uptake included concerns about possible side effects (56%) and a lack of sufficient knowledge about PrEP (52%). Education status significantly influenced preferred sources of PrEP information; school-going individuals were more likely to prefer school visits (61% compared to 43% of non-school-going youth, p = 0.01) and social media platforms (38% vs. 24%, p = 0.04) as avenues for receiving information. Age also impacted PrEP usage and information source preferences. Younger individuals were less inclined to use PrEP due to the inconvenience of taking a pill daily (mean age 20.4 years vs. 19.2 years, p = 0.04) and demonstrated a greater desire to know where they could obtain the medication (mean age 19.8 years vs. 18.9 years, p = 0.005). Additionally, younger participants showed a preference for being reached through newspapers (mean age 18.8 years vs. 19.8 years, p = 0.002), whereas older individuals favored billboard advertisements (mean age 19.8 years vs. 19.1 years, p = 0.04) as effective communication methods for PrEP information dissemination (Tables 4 and 5).

### Preferred communication methods for disseminating HIV Prevention, testing, and treatment Information

Overall, radio was identified as the most preferred method for disseminating HIV-related information, with 73% of participants favoring it, followed by television at 41%, and magazines being the least preferred at 16%. When examining the impact of education status, non-school-going individuals showed a significantly higher preference for radio (85%) compared to their school-going counterparts (60%, p < 0.01). Additionally, non-school-going youth were more likely to prefer receiving information via SMS (37% versus 19%, p = 0.004) and television (50% versus 32%, p = 0.013) compared to those who were school-going. Gender differences were also observed in communication preferences; females demonstrated a greater preference for print magazines (21%) compared to males (10.3%, p = 0.046) (Table 6).

## Discussion

The primary objective of this study was to explore how education status, age, and gender influence awareness and use of HIV prevention methods, beliefs about HIV infection risk, preferred access points for PrEP, and effective communication methods for disseminating HIV-related information. We found a higher awareness on traditional HIV prevention methods (condom use, abstinence and being faithful) as compared to the newer biomedical methods (pre-exposure prophylaxis, post-exposure prophylaxis, vaginal ring). Barriers to utilization of the biomedical HIV prevention methods (PrEP) included a lack awareness and concerns about adverse effects. The preferred communication channels for disseminating information regarding HIV prevention methods among the study population included mass media channels such as radio, television and SMS.

This study identified condom use as the most recognized method of HIV prevention among young people, likely due to the nature of youth relationships characterized by short-term commitments, exploratory behaviors and a high peak of sexual activity[29]. In such contexts, abstinence or fidelity, may not practically align with the youths’ relationship patterns[30–32]. Notably, awareness of abstinence was more pronounced among school-going youth, which could be attributed to their greater access to structured health education programs within schools[33, 34]. Uganda lifted a parliamentary ban on sexual education in schools beyond abstinence in May 2018; however, the curriculum primarily focuses on cultural and religious values, which may limit the scope of information available to students[35].

Our findings emphasize the importance of programs being implemented by the time of writing like the National Comprehensive Condom Programming Strategy, which ensure timely access to condoms for out of school youth and other at-risk populations like fisher folk[36]. Given these insights, we recommend enhancing these programs to emphasize not only condom use but also the importance of abstinence and fidelity, and newer biomedical HIV prevention methods providing a more holistic approach to HIV prevention. A systematic review on implementation and evaluation of HIV prevention studies across sub-Saharan Africa found that the most impactful interventions combined multiple HIV prevention foci[37]. Additionally, in line with our findings and those of previous research that highlight increased HIV-related risky sexual behavior among non-school going youth[38, 39], expanding educational efforts to community centers, social networks, and other accessible avenues could enhance awareness and adoption of HIV prevention strategies within this group.

The majority (72%) of participants in the study felt confident they would not contract HIV, largely due to their reliance on abstinence and condom use as key prevention methods. This finding is consistent with research by Muravha and colleagues in peri-urban South Africa, where many youths also perceived their HIV risk as low, often attributing this to sexual inactivity[40]. Similarly, studies from Nigeria revealed that students[41] and in-school adolescents[30] viewed sexual abstinence as the most effective HIV prevention strategy. Furthermore, school-going individuals were more likely to believe that abstinence contributed to their perceived low risk of contracting HIV. Additionally, individuals with higher educational status are more likely to practice abstinence[42], as research by Kefale et al. indicates that educated individuals are 3.9 times more likely to possess comprehensive knowledge of HIV, which predicts their likelihood to choose abstinence[43]. However, while abstinence is indeed 100% effective in preventing sexual transmission of HIV, abstinence-only programs, those that solely advocate for abstinence without promoting safe sex, have generally shown limited success in reducing risky behaviors[44]. In contrast, “abstinence-plus” programs, which combine abstinence with safe-sex education (like condom use), have demonstrated greater success in reducing HIV risk behaviors, especially in high-income countries[45]. Uganda’s A-B-C strategy (Abstinence, Be faithful, and Condom use), which promoted delayed sexual initiation, fewer partners, and condom use, in both school and community settings, similarly contributed to lowering HIV transmission rates[46].

We found a clear preference for accessing PrEP through private doctors and hospitals, with barriers to PrEP uptake including concerns about possible side effects and a lack of sufficient information about PrEP. These findings could be attributed to the aforementioned lack of awareness on PrEP, youth perceptions of PrEP as suitable only for immoral or promiscuous populations[47], and the lack of youth friendly services may further affect access to PrEP[48]. A study done in South Africa reported that adolescent girls at community health centers experience long queues, lack of privacy, harsher treatment, and stigmatization when accessing these services, which reinforces their preference for private doctors and hospitals over community health workers for accessing PrEP[49]. These findings are significant for public health strategies aimed at increasing PrEP uptake and adherence. Aligning PrEP delivery methods with patient preferences, such as utilizing private healthcare settings and leveraging preferred information channels like schools and social media, can enhance the effectiveness of PrEP programs. Addressing the identified barriers through targeted educational campaigns is essential to demystify PrEP, alleviate fears regarding side effects, and provide accurate information about its benefits and usage. Healthcare provider training should focus on enhancing communication skills to effectively address patient concerns about side effects and the importance of adherence to PrEP regimens. These strategies are crucial for mitigating misconceptions and increasing the overall acceptance and utilization of PrEP among youth.

The study revealed that radio is the most preferred method for disseminating HIV prevention, testing, and treatment information, followed by television, and magazines being the least preferred. This preference hierarchy was significantly influenced by education status and gender. Non-school-going individuals demonstrated a markedly higher preference for radio, SMS, and television compared to their school-going counterparts. The predominant preference for radio and television among non-school-going youth can be attributed to limited access to smartphones and the inability to afford them, making radio the most accessible and inexpensive medium available for reaching large numbers[50]. Furthermore, the prohibition of phones in most schools in Uganda necessitates reliance on traditional mass media channels like radio and television for information dissemination [51].

These findings are consistent with previous studies conducted in Uganda, which identified mass media–including radio, television, and newspapers–as the primary sources of HIV-related information[52, 53]. Mass media has been recognized as a powerful tool for enhancing public awareness, attitudes, and practices regarding HIV/AIDS by reaching a broad audience and facilitating behavioral change[54–56]. Similarly, a study in South Africa demonstrated that television series significantly contributed to HIV/AIDS knowledge, particularly in promoting HIV testing and preventive measures[57]. These studies collectively underscore the efficacy of mass media in public health education and interventions.

However, some studies highlighted that mass media can inadvertently widen the knowledge gap between educated and uneducated populations, particularly in rural areas with limited media accessibility[58]. This supports the knowledge gap hypothesis highlighting that the effectiveness of mass media as a communication tool may vary based on socioeconomic contexts and specific target populations.

This knowledge gap hypothesis is especially crucial when evaluating the current HIV prevention strategies. For example, biomedical methods like PreP are less likely to be known by people of low education status[59, 60]. Although mass media campaigns may rise general awareness, they risk deepening disparities in HIV knowledge and prevention behaviors, especially in rural and less educated populations.

Tailoring communication strategies to address the unique preferences of various demographic groups, such as integrating SMS campaigns for non-school-going youth and print materials for females, could help address these disparities. Furthermore, context-sensitive tools like community-driven interventions, localized health education, community radio and visual content for low-literacy populations show promise in bridging these knowledge gaps[8, 61, 62]. Addressing barriers to media access, such as affordability and regulatory restrictions, is essential to ensure equitable information dissemination across all population segments. Future research should explore the long-term impact of these communication methods on HIV-related behaviors, with a particular focus on rural Uganda and similar settings across sub-Saharan Africa.

### Strengths and limitations

This study includes a diverse sample of adolescents and young adults aged 15 to 24 years from both school-going and non-school-going populations. Recruitment from publicly funded schools and local communities in Rubirizi district captures a broad spectrum of youth perspectives on HIV prevention. This approach allows for meaningful comparisons based on education status, gender, and age, and could be expanded to larger populations for deeper insights into HIV prevention awareness and behaviors.

However, the study also has limitations.. First, its cross-sectional design limits the ability to infer causality or temporal relationships between the studied variables. As data were collected at a single point in time, it is not possible to determine changes in awareness, beliefs, or behaviors over time or to assess the impact of interventions. However, advanced analytical methods, alternative observational designs or longitudinal studies can sometimes offer insights into causality, a point that could be explored in future research. Second, the sampling of non-school-going youth with guidance of village health workers and snowball sampling, mayhave introduced selection bias. Participants recruited through social networks may share similar characteristics or behaviors, potentially limiting the sample diversity generalizability of study findings.

Third, self-reported data may be affected by information bias, including social desirability and recall biases. Participants may have over reported socially acceptable behaviors, such as abstinence or condom use, and underreported behaviors perceived as risky or stigmatized. Forth, the questionnaire was only pretested among school-going youth and only adjusted to ensure relevance for non-school-going youth. Lastly, the study was conducted in just one rural district with unique socio-cultural and economic contexts, which may limit the applicability of the findings to urban settings.

## Conclusion and recommendation

This study highlights how education status, age, and gender influence the HIV prevention awareness among youth. School going youth had greater awareness of biomedical and behavioral prevention methods likely due to exposure to structural health programs, while males had greater awareness to certain prevention methods, which may be influenced by gender norms and differences in health information access. Additionally, younger youth were more likely to practice abstinence. While the role of education in HIV prevention is well established, our study highlights the extent of the disparities aforementioned in a rural Ugandan setting and underscores the need for tailored interventions especially for non-school-going youth and address gender-based differences in knowledge and prevention uptake among vulnerable youth populations.

## Figures and Tables

**Table 1:** Characteristics of study participants

Characteristics	Description	Total (N)	Proportion (%)
**Age**	Mean ± SD	19.28 ± 2.32	
**Gender**	Female	109	50.46
	Male	107	49.54
**Relationship Status**	In a sexual relationship	29	13.43
	Not in a relationship	187	86.57
**Member of a Social Club**	Yes	131	60.65
	No	85	39.35
**House Description**	Mud and poles	33	15.28
	Brick House	183	84.72
**Went Hungry in the Last 10 Months**	Yes	82	38
	No	134	62
**Reason for Going Hungry** *(N = 82)*	No money to buy food	52	63.41
	No food	22	26.83
	Dieting	3	3.66
	Other reason	5	6.1
**Attitudes Towards PLHIV**	Positive	131	71.98
	Negative	51	28.02
**Media Use**	Uses radio/TV	167	77.31
	Uses print media	100	46.3
	Uses WhatsApp	71	32.81
	Uses internet for social networking sites	77	35.65
**Ever Had an HIV Test**	Yes	173	80.09
	No	43	19.91
**Thought They Will Get Infected with HIV**	Yes	61	28.24
	No	155	71.76
**Attitudes Towards Condom Use**	Positive	154	87
	Negative	23	13
Heard About Pre-Exposure Prophylaxis	Yes	95	43.98
	No	121	56.02

**Table 2:** Knowledge of Specific HIV Prevention Methods by Education Status, Gender, and Age

Prevention Method	Response	Non-School Going (n, %)	p-value	Female (n, %)	p-value	Age (mean ± SD)	p-value	Total (n, %)
**It can’t be prevented**	No	98 (90.7)	0.806	103 (94.5)	0.203	19.3 ± 2.3	0.775	198 (91.7)
	Yes	10 (9.3)		6 (5.5)		19.1 ± 2.7		18 (8.3)
**Using condoms**	No	36 (33.3)	0.297	34 (31.2)	0.72	19.0 ± 2.6	0.203	64 (29.6)
	Yes	72 (66.7)		75 (68.8)		19.4 ± 2.1		152 (70.4)
**Sticking to one sex partner**	No	69 (63.9)	0.485	74 (67.9)	0.054	19.1 ± 2.3	0.268	132 (61.1)
	Yes	39 (36.1)		35 (32.1)		19.5 ± 2.3		84 (38.9)
**Being faithful to one sex partner who is also faithful to you**	No	65 (60.2)	**0.001**	61 (56.0)	**0.041**	19.1 ± 2.3	0.28	105 (48.6)
	Yes	43 (39.8)		48 (44.0)		19.4 ± 2.3		111 (51.4)
**Reducing number of sex partners**	No	81 (75.0)	0.541	82 (75.2)	0.488	19.1 ± 2.2	0.13	157 (72.7)
	Yes	27 (25.0)		27 (24.8)		19.6 ± 2.5		59 (27.3)
**Abstaining from sex**	No	74 (68.5)	**<0.001**	51 (46.8)	1	19.7 ± 2.4	**0.017**	101 (46.8)
	Yes	34 (31.5)		58 (53.2)		18.9 ± 2.2		115 (53.2)
**Avoiding contact with blood**	No	81 (75.0)	**<0.001**	72 (66.1)	0.056	19.3 ± 2.3	0.865	128 (59.3)
	Yes	27 (25.0)		37 (33.9)		19.2 ± 2.3		88 (40.7)
**Using drugs to prevent the mother giving the child HIV**	No	81 (75.0)	**0.002**	79 (72.5)	**0.018**	19.3 ± 2.3	0.759	139 (64.4)
	Yes	27 (25.0)		30 (27.5)		19.2 ± 2.3		77 (35.6)
**Male circumcision**	No	89 (82.4)	**0.008**	84 (77.1)	0.392	19.3 ± 2.3	0.436	160 (74.1)
	Yes	19 (17.6)		25 (22.9)		19.1 ± 2.2		56 (25.9)
**Microbicides (gel/ring inserted into the vagina)**	No	95 (88.0)	0.5	101 (92.7)	0.242	19.2 ± 2.2	0.313	194 (89.8)
	Yes	13 (12.0)		8 (7.3)		19.7 ± 2.8		22 (10.2)
**Pre-exposure prophylaxis (PrEP)**	No	94 (87.0)	1	97 (89.0)	0.643	19.2 ± 2.3	0.088	189 (87.5)
	Yes	14 (13.0)		12 (11.0)		20.0 ± 2.4		27 (12.5)
Post-exposure prophylaxis (PEP)	No	98 (90.7)	1	100 (91.7)	0.614	19.3 ± 2.2	0.398	195 (90.3)
	Yes	10 (9.3)		9 (8.3)		18.9 ± 2.7		21 (9.7)

**Table 3:** Reasons for Low Perceived Risk of HIV Infection by Education Status, Gender, and Age

Reason	Level	Non-School Going (n, %)	p-value	Female (n, %)	p-value	Age (mean ± SD)	p-value	Total (n, %)
**Never had sex before**	No	54 (100.0)	0.769	77 (97.5)	0.494	18.9 ± 2.1	0.056	153 (98.7)
	Yes	0 (0.0)		2 (2.5)		16.0 ± 1.4		2 (1.3)
**Abstain/Withhold from Sex**	No	40 (74.1)	**<0.001**	39 (49.4)	0.801	19.4 ± 2.5	**0.002**	74 (47.7)
	Yes	14 (25.9)		40 (50.6)		18.3 ± 1.6		81 (52.3)
**Faithful to Partner**	No	28 (51.9)	**<0.001**	57 (72.2)	1	18.6 ± 1.9	**0.012**	111 (71.6)
	Yes	26 (48.1)		22 (27.8)		19.5 ± 2.5		44 (28.4)
**Trust My Partner**	No	42 (77.8)	0.655	62 (78.5)	0.623	18.7 ± 2.0	0.326	125 (80.6)
	Yes	12 (22.2)		17 (21.5)		19.2 ± 2.4		30 (19.4)
**Use Condoms**	No	27 (50.0)	**0.02**	57 (72.2)	**0.029**	18.8 ± 2.1	0.996	98 (63.2)
	Yes	27 (50.0)		22 (27.8)		18.8 ± 2.1		57 (36.8)
**Know My HIV Status**	No	39 (72.2)	0.352	61 (77.2)	1	18.6 ± 1.9	**0.022**	120 (77.4)
	Yes	15 (27.8)		18 (22.8)		19.5 ± 2.5		35 (22.6)
**Know the HIV Status of My Partner**	No	42 (77.8)	0.271	61 (77.2)	0.068	18.9 ± 2.1	0.389	129 (83.2)
	Yes	12 (22.2)		18 (22.8)		18.5 ± 2.0		26 (16.8)
**Do Not Have Sex with Sex Workers/Prostitutes**	No	48 (88.9)	0.814	74 (93.7)	**0.024**	19.0 ± 2.2	**0.026**	135 (87.1)
	Yes	6 (11.1)		5 (6.3)		17.9 ± 1.5		20 (12.9)
**Protected by Ancestors**	No	53 (98.1)	0.817	78 (98.7)	0.34	18.8 ± 2.1	0.295	150 (96.8)
	Yes	1 (1.9)		1 (1.3)		19.8 ± 1.6		5 (3.2)

**Table 4:** Preferred Access Points for PrEP by Education Status, Gender, and Age

Access Point	Response	Non-School Going (n, %)	p-value	Female (n, %)	p-value	Age (mean ± SD)	p-value	Total (n, %)
**Private doctor**	No	27 (54.0)	1	17 (50.0)	0.646	20.1 ± 2.3	0.139	43 (54.4)
	Yes	23 (46.0)		17 (50.0)		19.3 ± 2.3		36 (45.6)
**Hospital**	No	26 (52.0)	0.969	18 (52.9)	1	19.6 ± 2.6	0.579	42 (53.2)
	Yes	24 (48.0)		16 (47.1)		19.9 ± 2.1		37 (46.8)
**Clinic**	No	45 (90.0)	0.173	28 (82.4)	0.832	19.9 ± 2.4	0.243	67 (84.8)
	Yes	5 (10.0)		6 (17.6)		19.0 ± 1.7		12 (15.2)
**Pharmacy/Chemist**	No	44 (88.0)	1	30 (88.2)	1	19.8 ± 2.3	0.811	70 (88.6)
	Yes	6 (12.0)		4 (11.8)		19.6 ± 3.1		9 (11.4)
**Option School**	No	47 (94.0)	0.445	32 (94.1)	0.682	19.9 ± 2.3	0.06	72 (91.1)
	Yes	3 (6.0)		2 (5.9)		18.1 ± 2.4		7 (8.9)
**Community health workers**	No	47 (94.0)	0.463	32 (94.1)	0.804	19.7 ± 2.3	0.843	76 (96.2)
	Yes	3 (6.0)		2 (5.9)		20.0 ± 3.6		3 (3.8)
**Non-governmental organization (NGO)**	No	47 (94.0)	0.793	33 (97.1)	0.353	19.8 ± 2.3	0.802	73 (92.4)
	Yes	3 (6.0)		1 (2.9)		19.5 ± 2.6		6 (7.6)
**Family member**	No	49 (98.0)	1	33 (97.1)	0.887	19.8 ± 2.3	0.244	78 (98.7)
	Yes	1 (2.0)		1 (2.9)		-		1 (1.3)

**Table 5:** Reasons for Not Using PrEP by Education Status, Gender, and Age

Reason for Not Using PrEP	Response	Non-School Going (n, %)	p-value	Female (n, %)	p-value	Age (mean ± SD)	p-value	Total (n, %)
**Possible side effects**	No	37 (43.0)	0.827	32 (42.7)	0.799	19.5 ± 2.4	0.364	67 (44.4)
	Yes	49 (57.0)		43 (57.3)		19.2 ± 2.3		84 (55.6)
**I don’t know enough about it**	No	41 (47.7)	0.98	38 (50.7)	0.686	19.6 ± 2.4	0.268	73 (48.3)
	Yes	45 (52.3)		37 (49.3)		19.1 ± 2.2		78 (51.7)
I wouldn’t want my family or friends to know that I’m using it	No	73 (84.9)	1	62 (82.7)	0.468	19.4 ± 2.4	0.516	129 (85.4)
	Yes	13 (15.1)		13 (17.3)		19.0 ± 2.1		22 (14.6)
**If I use it, I would be more sexually active**	No	75 (87.2)	0.9	65 (86.7)	0.779	19.5 ± 2.3	0.101	133 (88.1)
	Yes	11 (12.8)		10 (13.3)		18.5 ± 2.3		18 (11.9)
I don’t want to drink a pill every day	No	73 (84.9)	0.143	69 (92.0)	0.317	19.2 ± 2.2	**0.044**	134 (88.7)
	Yes	13 (15.1)		6 (8.0)		20.4 ± 3.0		17 (11.3)
**If I use it, I will have riskier sex… like not using a condom**	No	78 (90.7)	0.435	73 (97.3)	0.063	19.3 ± 2.3	0.1	140 (92.7)
	Yes	8 (9.3)		2 (2.7)		20.5 ± 2.9		11 (7.3)
**Other**	No	81 (94.2)	1	72 (96.0)	0.731	19.3 ± 2.3	0.907	143 (94.7)
	Yes	5 (5.8)		3 (4.0)		19.2 ± 2.1		8 (5.3)

**Table 6:** Preferred Access Points for Receiving HIV-Related Messages by Education Status, Gender, and Age

Access Point	Response	Non-School Going (n, %)	p-value	Female (n, %)	p-value	Age (mean ± SD)	p-value	Total (n, %)
**Radio**	No	16 (14.8)	**< 0.001**	27 (24.8)	0.488	19.0 ± 2.0	0.25	59 (27.3)
	Yes	92 (85.2)		82 (75.2)		19.4 ± 2.4		157 (72.7)
**Newspaper**	No	77 (71.3)	0.767	73 (67.0)	0.423	19.2 ± 2.3	0.471	151 (69.9)
	Yes	31 (28.7)		36 (33.0)		19.4 ± 2.4		65 (30.1)
**Short Message Service (SMS)**	No	68 (63.0)	**0.004**	74 (67.9)	0.2	19.1 ± 2.2	**0.043**	156 (72.2)
	Yes	40 (37.0)		35 (32.1)		19.8 ± 2.4		60 (27.8)
**WhatsApp**	No	86 (79.6)	1	87 (79.8)	0.944	19.3 ± 2.2	0.318	171 (79.2)
	Yes	22 (20.4)		22 (20.2)		19.0 ± 2.5		45 (20.8)
**Social Networking Sites (e.g., Facebook, Twitter)**	No	84 (77.8)	1	79 (72.5)	0.056	19.3 ± 2.2	0.989	169 (78.2)
	Yes	24 (22.2)		30 (27.5)		19.3 ± 2.5		47 (21.8)
**Television**	No	73 (67.6)	**0.013**	61 (56.0)	0.474	19.4 ± 2.2	0.204	127 (58.8)
	Yes	35 (32.4)		48 (44.0)		19.0 ± 2.4		89 (41.2)
**Print Magazines**	No	93 (86.1)	0.575	86 (78.9)	**0.046**	19.3 ± 2.2	0.883	182 (84.3)
	Yes	15 (13.9)		23 (21.1)		19.2 ± 2.6		34 (15.7)

## Data Availability

The datasets used and/or analyzed during the current study are available from the corresponding author upon reasonable request.

## Abbreviations

HIV/AIDS: Human Immunodeficiency Virus / Acquired Immune Deficiency Syndrome
PMTCT: Prevention of Mother-To-Child Transmission
PEP: Post-Exposure Prophylaxis
PrEP: Pre-Exposure Prophylaxis
SMS: Short Message Service
UNAIDS: Joint United Nations Programme on HIV/AIDS
WHO: World Health Organization
